# Burns Treated with Ammonia

**Published:** 1847-10

**Authors:** 


7. *Burns treated with Ammonia.*—M. Guérard, physician to the "*Hotel Dieu*," has used, for upwards of twenty years, a concentrated solution of ammonia* in burns of the first and second degrees. He has frequently happened to burn himself with charcoal, phosphorus, gunpowder, &c., and the immediate application of this remedy has always arrested any further development. When the ends of the fingers are burnt he plunges them in the liquid without admixture of water. If the seat of the burn was such as to prevent this immersion, he would cover it with a compress dipped in the ammonia, and would prevent its evaporation by covering it with dry cloth. In such cases it is necessary to repeat the application from time to time, whenever the heat or sensation of burning returns. As soon as the ammonia is applied the pain ceases, and the relief continues longer, in proportion to the strength of the solution. According to what M. Guérard has himself experienced he believes that the application should be continued at least an hour, in order to give permanent relief, after which the burn may be left without any further dressing. If the burn be extensive, one hour will not be sufficient, but then the patient will be apprised of it by the return of pain. M. Guérard does not believe this application adapted to cases in which the skin is removed. The pain is immediately relieved, no phlyctæna are developed, and the cuticle dries and finally falls off like parchment. It is well to observe that if the application has been made to an extensive surface, the compresses should be handled with forceps, for concentrated ammonia very rapidly vesicates the skin in the healthy state. The patient as well as dresser, should also avoid breathing the vapour, and the vessels used should be made either of tin or of earthenware, inasmuch as copper is readily acted upon by ammonia.

*Aqua Ammonia, we presume.—*Trans.*

The use of ammonia in burns is not new. Physicians have long since observed that it prevents in such cases the developement of inflammation. It has been seen, however, that it is especially for burns of small extent, and in which the skin is not excoriated, that M. Guérard advises the use of this caustic. Thus far we see no objection to recommending its trial to practitioners. As to burns involving a large surface, it requires more circumspection. There are efficacious means in more common use, such as the oleo-calcareous liniment and carded cotton, prolonged cold baths and fomentations with iced water. There is at this time a case at the "*Hospital St. Louis*," in which the most happy results have been obtained with cold water.—Translated from Journ. des Conn. Méd-Chir.—Bull. Gén. de Therap., April, 1847, for *Ibid.*

